# Correction to “Pseudo‐mediastinal tumor: Brachiocephalic artery buckling”

**DOI:** 10.1002/ccr3.9350

**Published:** 2024-08-20

Kano Y, Murata K. Pseudo‐mediastinal tumor: Brachiocephalic artery buckling. *Clin Case Rep*. 2024;12(6):e8839

In the article entitled “Pseudo‐mediastinal tumor: Brachiocephalic artery buckling,” Figure 1B was incorrect. We hereby present the correct version of Figure 1.

**FIGURE 1:**
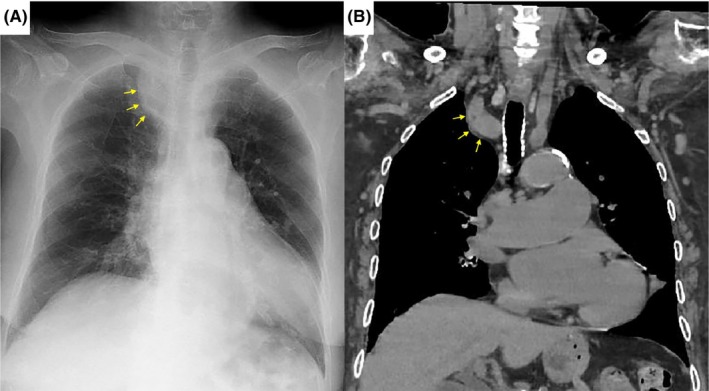
(A) Chest x‐ray demonstrating a mass‐like density in the right apex (arrows) resembling a mediastinal tumor but containing findings that are clues to the correct diagnosis: a positive extrapleural sign, negative tracheal deviation, and smooth margins. (B) Computed tomography (coronal image) revealing that the mediastinal shadow was in fact buckling of the brachiocephalic artery.

We apologize for this error.

